# Overview of the structure-based non-genomic effects of the nuclear receptor RXRα

**DOI:** 10.1186/s11658-018-0103-3

**Published:** 2018-08-07

**Authors:** Liqun Chen, Lingjuan Wu, Linyan Zhu, Yiyi Zhao

**Affiliations:** College of Biological Science and Engineering, Fuzhou University, Fuzhou, 350108 China

**Keywords:** Nuclear receptor, RXRα, Non-genomic action, Modification, Structure, RCSB protein data Bank

## Abstract

The nuclear receptor RXRα (retinoid X receptor-α) is a transcription factor that regulates the expression of multiple genes. Its non-genomic function is largely related to its structure, polymeric forms and modification. Previous research revealed that some non-genomic activity of RXRα occurs via formation of heterodimers with Nur77. RXRα–Nur77 heterodimers translocate from the nucleus to the mitochondria in response to certain apoptotic stimuli and this activity correlates with cell apoptosis. More recent studies revealed a significant role for truncated RXRα (tRXRα), which interacts with the p85α subunit of the PI3K/AKT signaling pathway, leading to enhanced activation of AKT and promoting cell growth in vitro and in animals. We recently reported on a series of NSAID sulindac analogs that can bind to tRXRα through a unique binding mechanism. We also identified one analog, K-80003, which can inhibit cancer cell growth by inducing tRXRα to form a tetramer, thus disrupting p85α–tRXRα interaction. This review analyzes the non-genomic effects of RXRα in normal and tumor cells, and discusses the functional differences based on RXRα protein structure (structure source: the RCSB Protein Data Bank).

**Electronic supplementary material:**

The online version of this article (10.1186/s11658-018-0103-3) contains supplementary material, which is available to authorized users.

## Introduction

Nuclear receptors (NRs) are a group of transcription factors that are widely distributed in the body and can be expressed in the cytoplasm or nucleus. There are more than 200 known NR proteins and they are involved in a range of biological processes, including proliferation, differentiation, apoptosis, metabolism, migration, inflammation and immune responses [1, 2]. Disorders in the expression of NRs or their regulated genes can lead to conditions such as cardiovascular disease, diabetes, obesity, reproductive system diseases, inflammation, cancer and Alzheimer’s disease [3–6].

RXRα (retinoid X receptor α; NR2B1), RXRβ (retinoid X receptor β; NR2B2) and RXRγ (retinoid X receptor γ; NR2B3) form a subgroup (the RXRs) belonging to the nonsteroidal receptor family of the NR superfamily [3]. The RXRs interact with ligands and co-regulators to regulate the coordinated expression of genes and thus play an important role in cell growth, development, homeostasis and many other physiological processes in the body [7–9].

RXRα, like the vast majority of NRs, consists of three distinct domains: an N-terminal A/B region that contains the ligand-independent activation domain AF1 (activation function-1); a DNA-binding domain (DBD); and a C-terminal ligand-binding domain (LBD) that contains the ligand-dependent activation domain AF2 (activation function-2). The LBD of RXRα also contains a canonical ligand-binding pocket (LBP), a co-regulator-binding surface groove and a dimerization surface [10–12].

RXRα regulates target gene transcription as a homodimer or heterodimer. RXRα homodimerizes with itself or heterodimerizes with many other nuclear receptors including peroxisome proliferator-activated receptor (PPAR), retinoic acid receptor (RAR), vitamin D receptor (VDR), thyroid hormone receptor (TR), liver X receptor (LXR), farnesoid X receptor (FXR), pregnane X receptor (PXR), chicken ovalbumin upstream promoter-transcription factor II (COUP-TFII) and nerve growth factor-induced gene B (Nur77, NGFI-B) [9, 13–16].

9-*cis*-RA was the first identified endogenous ligand of RXRα. Many other small molecules have been identified that bind to RXRα and modulate its activities [17–19]. RXRα and its partners act as ligand-dependent transcription factors through binding to specific DNA-response elements of the target genes [20, 21]. After ligand binding, a conformational change in RXRα triggers a cascade of events, such as co-activator or co-regulator recruitment, leading to positive or negative transcription activities and subsequent exertion of different biological functions [22].

Accumulating evidence indicates that RXRα also has extranuclear non-genomic functions aside from its genomic function in DNA binding and transactivation [23–26]. RXRα migration from the nucleus to the cytoplasm is correlated to cell survival, differentiation, inflammation and apoptosis. The non-genomic actions of RXRα are predominantly due to its cleavage, modification and polymeric forms.

In this review, we will briefly introduce the functions of RXRα in some biological processes, discuss its modulation and non-genomic action based on recent research, and summarize the RXRα structures from the RCSB Protein Data Bank [27–29].

## RXRα functions in various biological processes

The results of numerous experiments and biological analyses demonstrated that RXRα participates in a range of physiological processes. Altered expression of RXRα is involved in the development of many diseases. Genetic approaches in animals have shown that RXRα knockout is lethal at the embryonic stage [30, 31]. Active RXRα is required for ocular morphogenesis and the late steps in trophoblast differentiation [30, 31]. A reduction of RXRα expression in different tissues, such as the skin [32], adipose tissue [33], prostate tissue [34] and hepatocytes [35], may lead to various phenotypic changes, indicating that RXRα plays an irreplaceable role in these tissues.

Homodimerization of RXRα with itself or heterodimerization of RXRα with other nuclear receptors also affect its biological function. The phenotypes observed in most RXRα-knockout mice may be related to alterations in pathways regulated by its heterodimerization partners. RXRs heterodimerization with RARs is instrumental to retinoic acid signaling during embryonic development. Research has shown that binding of RXRα to PML–RARα is essential in the development of acute promyelocytic leukemia in transgenic mice, further illustrating the carcinogenicity of RXRα when it functions inappropriately [36]. RXRα–LXR heterodimers participate in AP1 signaling in keratinocytes [37]. Recent findings indicate that an N-terminally truncated form of RXRα (tRXRα) produced in cancer cells resides in the cytoplasm, where it promotes the growth of tumor cells [38–43]. Proteolytic cleavage of RXRα, which could reduce RXRα expression or enhance truncated RXRα expression in tumor cells, is also correlated with the development of certain malignancies [38, 39]. Different subcellular localization or nucleus-to-cytoplasm shuttle of RXRα may also affect the development of cancer and certain diseases. In addition, changes in RXRα function through phosphorylation, acetylation, ubiquitination and SUMOylation are associated with the development of human diseases.

## Modifications of RXRα

Limited proteolytic cleavage of RXRα protein has been found in many tumor cells [38–56]. Matsushima-Nishiwaki et al. found that RXRα was cleaved into tRXRα by m-calpain in HuH7 hepatocellular carcinoma (HCC) cells [38, 39]. Nomura et al. showed that in human placental choriocarcinoma JEG-3 cells, RXRα was cleaved into a 44-kDa tRXRα by the lysosomal enzyme cathepsin L-type protease at the RXRα N-terminal A/B region [40]. A 47-kDa tRXRα and 44-kDa tRXRα were detected in prostate cancer cell lines [41]. Recent studies showed that tRXRα is produced in many kinds of cancer cells and is detected in tumor tissues but not in normal tissues or tissues surrounding tumors in the same cancer patients [42–44]. Zhou et al. found that there is an N-terminal deletion of RXRα that lacks 80 amino acids. This tRXRα (RXRα-Δ80) can interact with the p85α subunit of the PI3K/AKT survival pathway and promote cancer cell proliferation in the majority of cancer cells [42]. In our study, tRXRα was detected in the cytoplasm while RXRα was detected in the nucleus [43]. We also found extensive intramolecular interaction between the N terminus and the C terminus (N/C) of full-length RXRα but not that of RXRα-Δ80, which explains why tRXRα can interact with p85α while full-length RXRα cannot [43]. The results of our study suggested that amino acids from 60 to 80 are critical for the RXRα N/C interaction. The N/C intramolecular interaction involves the N-terminal A/B domain and the C-terminal AF2/H12 [43]. Gao et al. identified a truncated RXRα that lacks 90 N-terminal amino acids and can activate AKT when overexpressed in cancer cells. They also investigated the role of calpain II in producing this kind of tRXRα [44]. Moreover, glycogen synthase kinase 3 beta (GSK-3β) can negatively regulate tRXRα production by inhibiting calpainII expression [44].

The N-terminal A/B domains of RXRα contain many phosphorylation sites, including serine 61, serine 75, threonine 87. Apoptosis is induced when hyperphosphorylation happened at these sites [45]. Serine 260 of RXRα, a consensus phosphorylation site of mitogen-activated protein kinase, is closely linked to RXRα-retarded degradation and the promotion of cancer cell growth in human HCC-derived HuH7 cells [46]. In addition, the non-genomic actions of RXRα also involve the inhibition of c-Jun N-terminal kinase (JNK) activation/phosphorylation and subsequent c-Jun phosphorylation. RXRα undergoes rapid post-translational modifications, including JNK-mediated phosphorylation, which correlates with a reduction in RXRα function [47–51].

Kopf et al. showed that in F9 murine embryonal carcinoma cells and transfected COS-1 African green monkey kidney fibroblast cells, nuclear retinoid receptors such as RARα1 (retinoic acid receptor α1), RARγ2 (retinoic acid receptor γ2), and RXRα1 (retinoid X receptor α1) are degraded in a retinoic acid-dependent manner through the ubiquitin-proteasome pathway [52]. Aguirre et al. reported that lipopolysaccharide, tumor necrosis factor α (TNFα) and interleukin-1β rapidly and substantially stimulate SUMOylation of RXRα in human hepatocellular carcinoma HuH-7 cells, indicating that SUMOylation of RXRα is involved in the inflammatory signaling pathways [53]. Another study showed that p300 can induce acetylation of RXRα at RXRα lysine 145 (K145) [54]. The orphan nuclear receptor Nur77 exerts a negative regulation on p300-induced RXRα acetylation [54–56].

## Different polymeric forms of RXRα

RXRα can form heterodimers with many nuclear receptors to assist in nucleus-to-cytoplasm transfer. Cao et al. found that in response to apoptotic stimuli, TR3 translocates from the nucleus to the mitochondria to interact with Bcl-2 and induce cytochrome c release, ultimately leading to cell apoptosis [57]. Mitochondrial targeting of TR3, but not its DNA binding and transactivation, is essential for its pro-apoptotic effect [55, 57, 58]. RXRα is required for the nuclear export and mitochondrial targeting of Nur77 through their unique heterodimerization. The effects of RXRα are attributed to a putative nuclear export sequence (NES) in its carboxyl-terminal region [57]. Interestingly, when treated with 9-*cis*-RA (the natural ligand of RXRα), RXRα is translocated with TR3 from the nucleus to the mitochondria, and apoptosis is induced [54, 55].

Zeng et al. revealed that an extract of *Hypericum sampsonii* had a remarkable effect on RXRα subcellular localization in various cancer cells [59]. Treatment of NCI-H460 human non-small cell lung cancer cells with *H. sampsonii* extract resulted in relocalization of RXRα from the nucleus to the cytoplasm, where it associated with mitochondria, leading to cytochrome c release and apoptosis. *H. sampsonii* extract effectively inhibits the growth of cells of various cancer cell lines, including H460 lung cancer, MGC-803 stomach cancer and SMMC7721 liver cancer. *H. sampsonii* fails to inhibit the growth of CV-1 African green monkey kidney fibroblast cells, which lack detectable RXRα, but transfection of RXRα into CV-1 cells restores the apoptotic response to *H. sampsonii*. This interesting phenomenon suggests that the growth-inhibiting effect of *H. sampsonii* extract depends on the RXRα levels. Furthermore, the apoptotic effect of *H. sampsonii* is significantly enhanced when RXRα is overexpressed in H460 cells. These results demonstrate that subcellular localization of RXRα is modulated by *H. sampsonii* which contains ingredient(s) that can induce apoptosis of cancer cells.

Another study showed that RXRα can assist ERΔDBD and ERΔHinge translocation from the nucleus to the cytoplasm. After treatment with E2 (steroid hormone 17β-estradiol), ERΔDBD returns to the nucleus while the ERΔHinge remains in the cytoplasm [60]. In studying the regulation of RARγ subcellular localization, Yan et al. observed that ectopically overexpressed RARγ is mainly cytoplasmic irrespective of serum concentration and cell density [61]. The cytoplasmic retention of RARγ is inhibited by the ligand all-trans-retinoic acid (ATRA). In addition, co-expression of RXRα results in nuclear localization of RARγ through their heterodimerization [62]. The nuclear receptor PPARγ (peroxisome proliferator-activated receptor γ) is a key regulator of glucose homeostasis and insulin sensitization. It must heterodimerize with its dimeric partner, RXR, to bind DNA and associated coactivators such as p160 family members or PGC-1α [63]. Xu and Zeng found that the compound Z-10, a nitro-ligand of RXRα [64, 65], induces PML-RARα cleavage and APL cell apoptosis by disrupting PML–RARα–RXRα complexes in a cAMP-independent manner. RXRα is vital for the stability of both PML–RARα and RARα, likely through direct interactions. The binding of compound Z-10 to RXRα dramatically inhibits the interaction of RXRα with PML–RARα but not that with RARα, leading to Z-10’s selective induction of PML–RARα but not RARα degradation. 1α, 25(OH)2D3 binds to the vitamin D receptor, which belongs to the NR family. This forms a complex with RXR to regulate gene expression. An interaction between RXR and VDR polymorphisms has been demonstrated, indicating that they have an impact on the risk of ovarian cancer [66, 67].

Besides heterodimers and its homodimer, RXRα can also form a tetramer. Zhang et al. identified danthron, which is extracted from the traditional Chinese medicine rhubarb, as a specific RXRα antagonist [68]. Danthron can bind to the tetrameric RXRα LBD in a specific stoichiometric ratio, and such binding can influence co-repressor SMRT affinity to the receptor. The determined crystal structure of danthron-soaked RXRα LBD suggests a new mechanism for danthron antagonism to tetrameric RXRα [68]. We solved the crystal structures to reveal that the non-steroidal anti-inflammatory drug (NSAID) sulindac analog K-8008 can bind to the RXRα LBD tetramer through a novel hydrophobic region that is located on the surface of the monomer and near the dimer–dimer interface in the tetramer. Unlike the binding of other published ligands, the binding of K-8008 does not change the shape of the apo RXRα LBP, i.e., K-8008 binding may help to stabilize the RXRα LBD tetramer [69]. We also reported on the crystal structure of RXRα LBD in complex with K-80003, which is also derived from sulindac, and characterized the role of K-80003-mediated tRXRα tetramerization in regulating its interaction with p85α and non-genomic activation of PI3K signaling. Our results revealed a previously unrecognized role for RXRα tetramers in modulating subcellular localization and non-genomic interaction with cytoplasmic signaling proteins. We also showed that K-80003 inhibits tRXRα interaction with p85α by stabilizing a tetrameric form of tRXRα through a ‘three-pronged’ mechanism involving both canonical and non-canonical binding [43].

In conclusion, our results elucidated a previously unrecognized role for RXRα tetramers and demonstrated that conformational selection plays a critical role in the regulation of the non-genomic function of RXRα. We showed that the tetramerization of RXRα can be regulated by several mechanisms including ligand binding, intra domain interactions and non-genomic interactions with cytoplasmic signaling proteins [43].

## Different binding sites of RXRα for drug targeting

Canonical ligands bind to RXRα LBP to directly mediate RXRα transcriptional activity [3, 7, 10]. 9-*cis*-RA was the first compound identified as a natural RXRα ligand that binds to RXRα LDP and alters the ligand-binding pocket conformation. In addition, the synthetic RXRα agonist SR11237 1,3-dioxalane ring and the 9-*cis*-RA 19-methyl group occupy the same region of RXRα LBP [10]. The RXR-based drug Targretin (bexarotene), which is approved by the FDA for treating cutaneous T-cell lymphoma (CTCL patients), selectively binds to RXRs and does not have significant RAR binding and transactivation activity [7]. Several dietary fatty acids, including oleic acid, docosahexaenoic acid (DHA) and phytanic acid, bind RXRα and act as natural RXRα ligands [10, 11]. In addition to the RXRα ligands mentioned above, numerous natural products and synthetic compounds (retinoids) have been reported to bind to the RXRα ligand-binding pocket and to modulate its activities [17–19, 70–76].

Apart from the canonical ligand-binding site, many new alternate binding sites have been reported for nuclear receptors in recent years. Among these, the co-regulator-binding site is the most studied. Recently, by employing a docking-based virtual screening approach, Chen et al. identified a new RXRα antagonist, named compound 23, which can target the co-regulator-binding site of RXRα [77]. The compound does not bind to the ligand-binding pocket but to a hydrophobic groove on the surface of RXRα, a region where the binding sites of co-repressor and co-activator overlap [77]. This compound can also suppress AKT activation and thereby promote apoptosis of cancer cells in an RXRα-dependent manner by inhibiting tRXRα interaction with the p85α subunit of PI3K in vitro and in animals. Compound 23 is the first example of an RXRα modulator that acts via the co-regulator-binding site rather than binding to the classical LBP of RXRα.

We identified two new compounds, K-8008 and K-8012, which are NSAID sulindac analogs that can bind to the hydrophobic region of RXRα LBD near the entry and the edge of the cognate LBP [69]. The hydrophobic region does not overlap with the binding region of 9-*cis*-RA on RXRα. This new binding pattern explains why K-8008 and K-8012 compounds fail to compete with the binding of 9-*cis*-RA but can still inhibit cancer cell growth [69]. K-80003, another NSAID sulindac analog, promotes tetramerization of tRXRα but not RXRα. We solved the crystal structure of RXRα LBD in complex with K-80003 to a resolution of 2.6 Å. We found that the RXRα LBD–K-80003 complex adopts a tetrameric structure. These ‘tetramer’ interfaces comprise three sub-regions: parallel packing between symmetry-related H3 helices; ‘end-to-end’ packing at H11 that reduces their length by two helical turns (compared with the agonist-bound structure); and the invasion of each H12 helix into its apposing domain, where it binds to the co-regulator-binding groove, consisting of elements of H3 and H4 [43].

## Summary of RXRα protein structures

The protein structure and formation of RXRα are very important for investigating its biological functions and developing RXRα-targeting drugs. Different structures and aggregation methods may lead to different forms of RXRα. The structure of RXRα is available from the RCSB Protein Data Bank (PDB; https://www.rcsb.org/) [29, 78–83].

Here, we organized and summarized the structures of the RXRα as reported in the PDB database, showing the 3D structures of RXRα complexed with DNA, the structures of RXRα with the other nuclear receptors, the structures of RXRα with various compounds and the structure of apo-RXRα (Tables 1, 2, 3, 4 and the Additional file 1: Table S1).

**Table 1:**
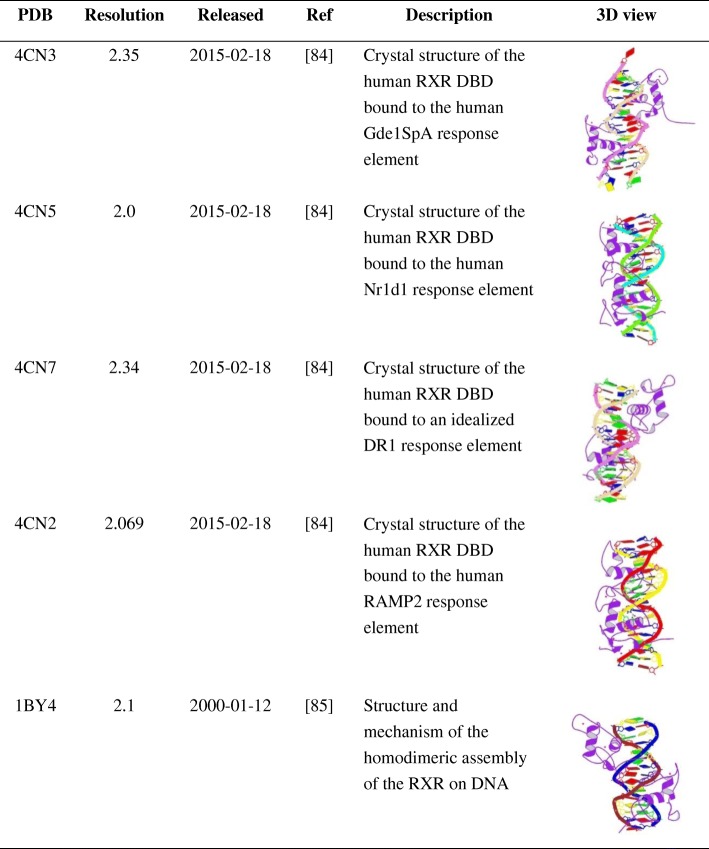
The 3D structure of RXRα and DNA [84, 85]

**Table 2:**
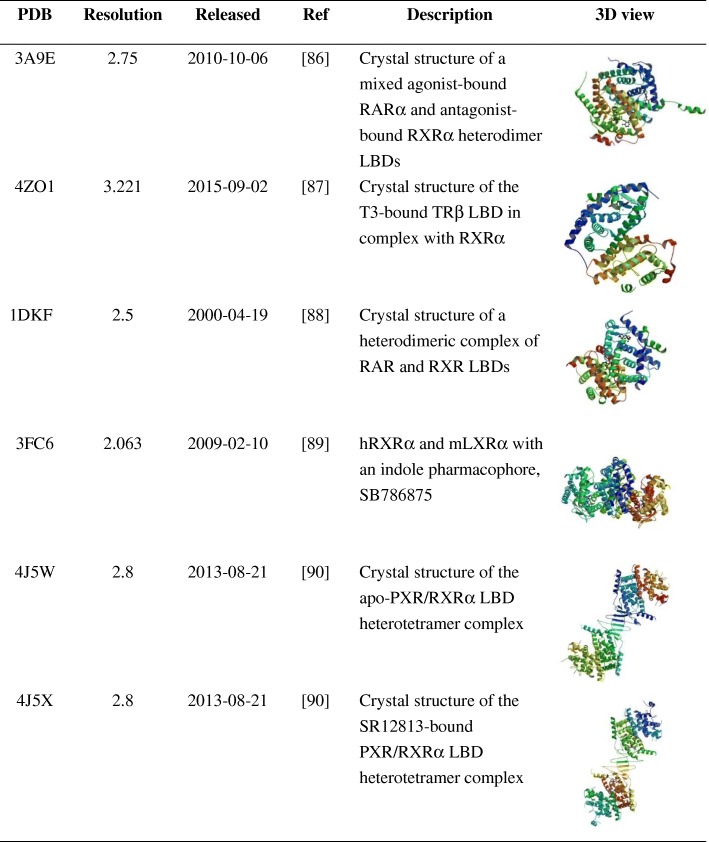
The 3D structure of RXRα with other nuclear receptors [86–90]

**Table 3:**
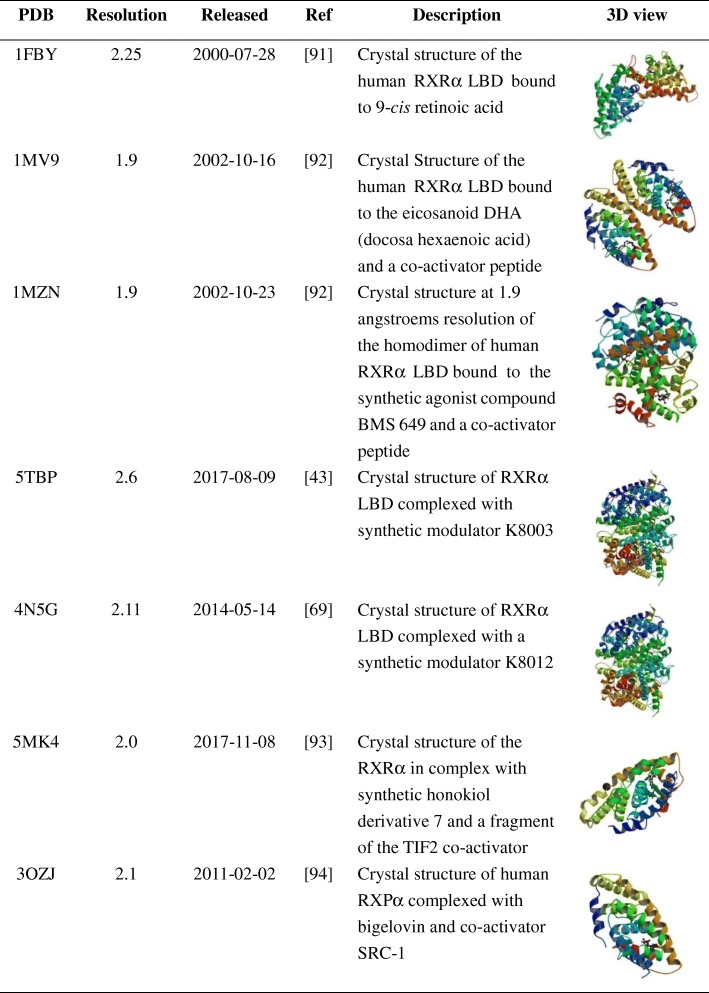
The 3D structure of RXRα with compounds [43, 69, 91–94]

**Table 4:**
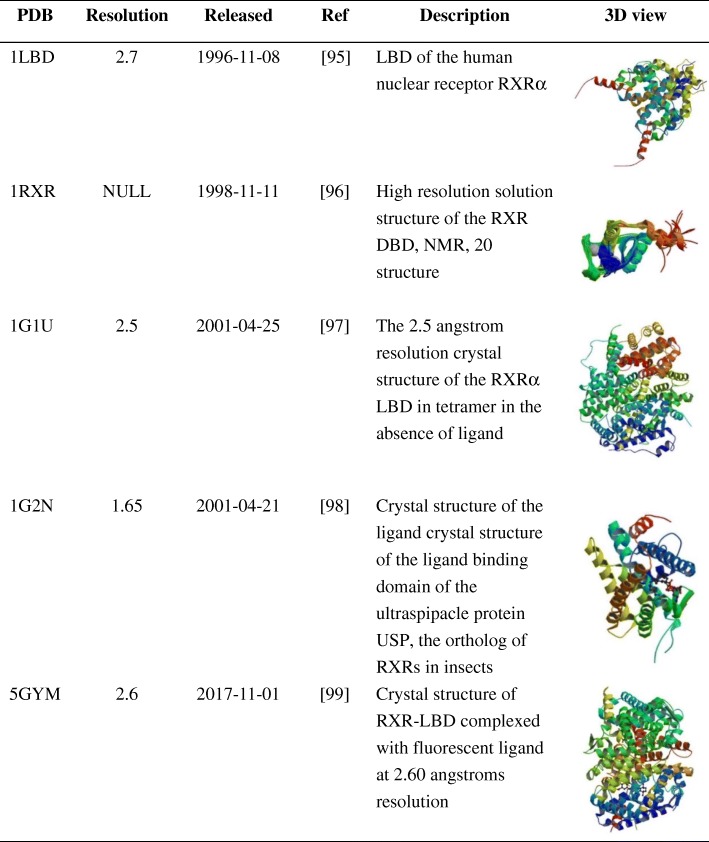
The 3D structure of RXRα [95–99]

## Conclusions and perspectives

RXRα is a nuclear receptor that regulates various biological effects such as cell growth, differentiation and apoptosis [3–9]. It plays a significant role in human physiology and pathology and can be regulated by endogenous and synthetic ligands and other small molecules. The continual discovery of new non-genomic actions of RXRα has greatly expanded our understanding of its cellular functions.

The effects of RXRα unrelated to its transcriptional activity have lately received increasing attention from researchers. Initially, it was noticed that upon certain stimuli, RXRα translocates NR4A1 from the nucleus to mitochondria and triggers apoptosis [56–59]. Direct interaction of tRXRα with the PI3K/AKT signaling subunit p85α may cause cancer cell growth and appears to be a potential target for anti-cancer drug development [42, 43, 69–73]. RXRα is unique in that it can be cleaved, phosphorylated, ubiquited, SUMOylated and acetylated, and it can form not only homodimers and heterodimers but also homotetramers, suggesting that the equilibrium between these different states plays a role in regulating RXRα functions [6–12, 41, 49, 53, 55, 57, 62].

RXRα and tRXRα are intriguing targets for pharmacological intervention, so it is of great importance to discovery new strategies for targeting them. The development of compounds or inhibitors targeting tRXRα by binding to a novel binding site may lead to a departure from the traditional approach of targeting LBP and initiate a new paradigm for targeting the RXRα surface for more effective and specific therapeutics [7, 10, 11, 69, 77].

Our analysis based on data from the PDB shows that currently known RXRα structures can be used for structural and functional predictions for new drug development. Further studies on RXRα, including its involvement in signaling pathways, its various structures, and targeted drug development are still needed. Hybrid approaches combining a variety of biophysical, biochemical, biomathematical, bioinformatic and modeling techniques will be used increasingly to predict and determine the structure-based functions of the protein.

Critically, while RXRα functions as a transcription factor in the nucleus, it can also directly interact with other nuclear receptors or proteins in different cellular compartments to exert multiple biological functions. This makes it a fascinating and important subject for further research using the described methods.

## Additional file

Additional file 1: **Table S1.** The 3D structure of RXRα with compounds. (DOCX 1855 kb)

## Abbreviations

AF1: Activation function-1
AF2: Activation function-2
CTCL: Cutaneous T-cell lymphoma
DBD: DNA-binding domain
DHA: Docosahexaenoic acid
E2: Steroid hormone 17β-estradiol
GSK-3β: Glycogen synthase kinase 3 beta
JNK: c-Jun N-terminal kinase
LBD: Ligand binding domain
LBP: Ligand-binding pocket
NES: Nuclear export sequence
NRs: Nuclear receptors
NSAID: Nonsteroidal anti-inflammatory drug
PPARγ: Peroxisome proliferator-activated receptor γ
RA: Retinoic acid
RARα1: Retinoic acid receptor α1
RARγ2: Retinoic acid receptor γ2
RCSB PDB: RCSB Protein Data Bank
RXR: Retinoid X receptor
RXRα: Retinoid X receptor-α
RXRα1: Retinoid X receptor α1
RXRβ: Retinoid X receptor β
RXRγ: Retinoid X receptor γ
TNFα: Tumor necrosis factor α
tRXRα: Truncated RXRα

## References

[1] Mckenna NJ (2011). Discovery-driven research and bioinformatics in nuclear receptor and coregulator signaling. Biochim Biophys Acta.

[2] Mangelsdorf DJ, Thummel C, Beato M, Herrlich P, Schütz G, Umesono K (1995). The nuclear receptor superfamily: the second decade. Cell.

[3] Evans RM, Mangelsdorf DJ (2014). Nuclear receptors, RXR, and the big bang. Cell.

[4] Altucci L, Leibowitz MD, Ogilvie KM, de Lera AR, Gronemeyer H (2007). RAR and RXR modulation in cancer and metabolic disease. Nat Rev Drug Discov.

[5] Germain P, Staels B, Dacquet C, Spedding M, Laudet V (2006). Overview of nomenclature of nuclear receptors. Pharmacol Rev.

[6] Olefsky JM (2001). Nuclear receptor minireview series. J Biol Chem.

[7] Zhang X, Zhou H, Su Y (2016). Targeting truncated RXRalpha for cancer therapy. Acta Biochim Biophys Sin.

[8] Zhang XK, Su Y, Chen LQ, Chen F, Liu J, Zhou H (2015). Regulation of the nongenomic actions of retinoid X receptor-alpha by targeting the coregulator-binding sites. Acta Pharmacol Sin.

[9] Szanto A, Narkar V, Shen Q, Uray IP, Davies PJA, Nagy L (2004). Retinoid X receptors: X-ploring their (patho)physiological functions. Cell Death Differ.

[10] Dawson MI, Xia ZB (2012). The retinoid X receptors and their ligands. Bba-Mol Cell Biol L.

[11] Dawson MI, Zhang XK (2002). Discovery and design of retinoic acid receptor and retinoid X receptor class- and subtype-selective synthetic analogs of all-trans-retinoic acid and 9-*cis*-retinoic acid. Curr Med Chem.

[12] Desvergne B (2007). RXR: from partnership to leadership in metabolic regulations. Vitam Horm.

[13] Lee KN, Jang WG, Kim EJ, Oh SH, Son HJ, Kim SH (2012). Orphan nuclear receptor chicken ovalbumin upstream promoter-transcription factor II (COUP-TFII) protein negatively regulates bone morphogenetic protein 2-induced osteoblast differentiation through suppressing runt-related gene 2 (Runx2) activity. J Bio Chem.

[14] Roszer T, Menendez-Gutierrez MP, Cedenilla M, Ricote M (2013). Retinoid X receptors in macrophage biology. Trends Endocrinol Metab.

[15] Ahuja HS, Szanto A, Nagy L, Davies PJ (2003). The retinoid X receptor and its ligands: versatile regulators of metabolic function, cell differentiation and cell death. J Biol Regul Homeost Agents.

[16] Tanaka T, De Luca LM (2009). Therapeutic potential of “rexinoids” in cancer prevention and treatment. Cancer Res.

[17] Kagechika H (2002). Novel synthetic retinoids and separation of the pleiotropic retinoidal activities. Curr Med Chem.

[18] Nagpal S, Chandraratna RA (2000). Recent developments in receptor-selective retinoids. Curr Pharm Des.

[19] Perez E, Bourguet W, Gronemeyer H, de Lera AR (1821). Modulation of RXR function through ligand design. Biochim Biophys Acta.

[20] Bastien J, Rochette-Egly C (2004). Nuclear retinoid receptors and the transcription of retinoid-target genes. Gene.

[21] Chandra V, Huang P, Hamuro Y, Raghuram S, Wang Y, Burris TP (2008). Structure of the intact PPAR-γ–RXR-α nuclear receptor complex on DNA. Nature.

[22] Westin S, Kurokawa R, Nolte RT, Wisely GB, Mcinerney EM, Rose DW (1998). Interactions controlling the assembly of nuclear-receptor heterodimers and co-activators. Nature.

[23] Casas F, Daury L, Grandemange S, Busson M, Seyer P, Hatier R (2003). Endocrine regulation of mitochondrial activity: involvement of truncated RXRalpha and c-Erb Aalpha1 proteins. FASEB J.

[24] Katagiri Y, Takeda K, Yu ZX, Ferrans VJ, Ozato K, Guroff G (2000). Modulation of retinoid signalling through NGF-induced nuclear export of NGFI-B. Nat Cell Biol.

[25] Ghose R, Zimmerman TL, Thevananther S, Karpen SJ (2004). Endotoxin leads to rapid subcellular re-localization of hepatic RXRalpha: a novel mechanism for reduced hepatic gene expression in inflammation. Nucl Recept.

[26] Moraes LA, Bishopbailey D, Gibbins JM (2007). Non-genomic signalling of the retinoic X receptor through inhibition of Gq signalling in human platelets. Heart..

[27] Berman HM, Westbrook J, Feng Z, Gilliland G, Bhat TN, Weissing H (2000). The protein data Bank, 1999–[J]. Int Tables Crystallogr.

[28] Zhang XK, Lehmann J, Hoffmann B, Dawson MI, Cameron J, Graupner G (1992). Homodimer formation of retinoid X-receptor induced by 9-*cis* retinoic acid. Nature.

[29] Rose PW, Prlic A, Bi CX, Bluhm WF, Christie CH, Dutta S (2015). The RCSB protein data Bank: views of structural biology for basic and applied research and education. Nucleic Acids Res.

[30] Mascrez B, Ghyselinck NB, Chambon P, Mark M (2009). A transcriptionally silent RXRalpha supports early embryonic morphogenesis and heart development. Proc Natl Acad Sci U S A.

[31] Mark M, Ghyselinck NB, Chambon P (2009). Function of retinoic acid receptors during embryonic development. Nucl Recept Signal.

[32] Li M, Indra AK, Warot X, Brocard J, Messaddeq N, Kato S (2000). Skin abnormalities generated by temporally controlled RXRalpha mutations in mouse epidermis. Nature.

[33] Takeshi I, Ming J, Pierre C, Daniel M (2001). Impaired Adipogenesis and lipolysis in the mouse upon selective ablation of the retinoid X receptor α mediated by a tamoxifen-inducible chimeric Cre recombinase (Cre-ERT2) in adipocytes. Proc Natl Acad Sci U S A.

[34] Huang J, Powell WC, Khodavirdi AC, Wu J, Makita T, Cardiff RD (2002). Prostatic intraepithelial neoplasia in mice with conditional disruption of the retinoid X receptor alpha allele in the prostate epithelium. Cancer Res.

[35] Wan YJ, An D, Cai Y, Repa JJ, Hung-Po CT, Flores M (2000). Hepatocyte-specific mutation establishes retinoid X receptor alpha as a heterodimeric integrator of multiple physiological processes in the liver. Mol Cell Biol.

[36] Martens JH, Brinkman AB, Simmer F, Francoijs KJ, Nebbioso A, Ferrara F (2010). PML-RARalpha/RXR alters the epigenetic landscape in acute promyelocytic leukemia. Cancer Cell.

[37] Shen Q, Bai Y, Chang KCN, Wang Y, Burris TP, Freedman LP (2011). Liver X receptor-retinoid X receptor (LXR-RXR) heterodimer cistrome reveals coordination of LXR and AP1 signaling in keratinocytes. J Biol Chem.

[38] Matsushima-Nishiwaki R, Shidoji Y, Nishiwaki S, Moriwaki H, Muto Y (1996). Limited degradation of retinoid x receptor by calpain. Biochem Biophys Res Commun.

[39] Matsushima-Nishiwaki R, Shidoji Y, Nishiwaki S, Yamada T, Moriwaki H, Muto Y (1996). Aberrant metabolism of retinoid X receptor proteins in human hepatocellular carcinoma. Mol Cell Endocrinol.

[40] Nomura Y, Nagaya T, Yamaguchi S, Katunuma N, Seo H (1999). Cleavage of RXRalpha by a lysosomal enzyme, cathepsin L-type protease. Biochem Biophys Res Commun.

[41] Zhong C, Yang S, Huang J, Cohen MB, Roy-Burman P (2003). Aberration in the expression of the retinoid receptor, RXRalpha, in prostate cancer. Cancer Biol Ther.

[42] Zhou H, Liu W, Su Y, Wei Z, Liu J, Kolluri SK (2010). NSAID sulindac and its analog bind RXRalpha and inhibit RXRalpha-dependent AKT signaling. Cancer Cell.

[43] Chen L, Aleshin AE, Alitongbieke G, Zhou Y, Zhang X, Ye X (2017). Modulation of nongenomic activation of PI3K signalling by tetramerization of N-terminally-cleaved RXRalpha. Nat Commun.

[44] Gao W, Liu J, Hu M, Huang M, Cai S, Zeng Z (2013). Regulation of proteolytic cleavage of retinoid X receptor alpha by GSK-3beta. Carcinogenesis.

[45] Tarrade A, Bastien J, Bruck N, Bauer A, Gianni M, Rochette-Egly C (2005). Retinoic acid and arsenic trioxide cooperate for apoptosis through phosphorylated RXR alpha. Oncogene.

[46] Matsushima-Nishiwaki R, Okuno M, Adachi S, Sano T, Akita K, Moriwaki H (2001). Phosphorylation of retinoid X receptor alpha at serine 260 impairs its metabolism and function in human hepatocellular carcinoma. Cancer Res.

[47] Zimmerman TL, Thevananther S, Ghose R, Burns AR, Karpen SJ (2006). Nuclear export of retinoid X receptor alpha in response to interleukin-1beta-mediated cell signaling: roles for JNK and SER260. J Biol Chem.

[48] Adam-Stitah S, Penna L, Chambon P, Rochette-Egly C (1999). Hyperphosphorylation of the retinoid X receptor alpha by activated c-Jun NH2-terminal kinases. J Biol Chem.

[49] Lee HY, Suh YA, Robinson MJ, Clifford JL, Hong WK, Woodgett JR (2000). Stress pathway activation induces phosphorylation of retinoid X receptor. J Biol Chem.

[50] Solomon C, White JH, Kremer R (1999). Mitogen-activated protein kinase inhibits 1,25-dihydroxyvitamin D3 dependent signal transduction by phosphorylating human retinoid X receptor alpha. J Clin Invest.

[51] Yoshimura K, Muto Y, Shimizu M, Matsushima-Nishiwaki R, Okuno M, Takano Y (2007). Phosphorylated retinoid X receptor alpha loses its heterodimeric activity with retinoic acid receptor beta. Cancer Sci.

[52] Kopf E, Plassat JL, Vivat V, de The H, Chambon P, Rochette-Egly C (2000). Dimerization with retinoid X receptors and phosphorylation modulate the retinoic acid-induced degradation of retinoic acid receptors alpha and gamma through the ubiquitin-proteasome pathway. J Biol Chem.

[53] Aguirre RS, Karpen SJ (2013). Inflammatory mediators increase SUMOylation of retinoid X receptor alpha in a c-Jun N-terminal kinase-dependent manner in human hepatocellular carcinoma cells. Mol Pharmacol.

[54] Zhao WX, Tian M, Zhao BX, Li GD, Liu B, Zhan YY (2007). Orphan receptor TR3 attenuates the p300-induced acetylation of retinoid X receptor-alpha. Mol Endocrinol.

[55] Wu Q, Lin XF, Ye XF, Zhang B, Xie Z, Su WJ (2004). Ubiquitinated or sumoylated retinoic acid receptor alpha determines its characteristic and interacting model with retinoid X receptor alpha in gastric and breast cancer cells. J Mol Endocrinol.

[56] Lin XF, Zhao BX, Chen HZ, Ye XF, Yang CY, Zhou HY (2004). RXRalpha acts as a carrier for TR3 nuclear export in a 9-*cis* retinoic acid-dependent manner in gastric cancer cells. J Cell Sci.

[57] Cao X, Liu W, Lin F, Li H, Kolluri SK, Lin B (2004). Retinoid X receptor regulates Nur77/TR3-dependent apoptosis [corrected] by modulating its nuclear export and mitochondrial targeting. Mol Cell Biol.

[58] Lee KW, Ma L, Yan X, Liu B, Zhang XK, Cohen P (2005). Rapid apoptosis induction by IGFBP-3 involves an insulin like growth factor-independent nucleomitochondrial translocation of RXRalpha/Nur77. J Biol Chem.

[59] Zeng JZ, Sun DF, Wang L, Cao X, Qi JB, Yang T (2006). Hypericum sampsonii induces apoptosis and nuclear export of retinoid X receptor-alpha. Carcinogenesis.

[60] Yang CY, Zhang XY, Wu Q (2007). Subcellular localization of estrogen receptor alpha and its correlation with cell proliferation. J Xiamen Univ (Natural Science).

[61] Yan TD, Wu H, Zhang HP, Lu N, Ye P, Yu FH (2010). Oncogenic potential of retinoic acid receptor-gamma in hepatocellular carcinoma. Cancer Res.

[62] Han YH, Zhou H, Kim JH, Yan TD, Lee KH, Wu H (2009). A unique cytoplasmic localization of retinoic acid receptor-gamma and its regulations. J Biol Chem.

[63] Osz J, Pethoukhov MV, Sirigu S, Svergun DI, Moras D, Rochel N (2012). Solution structures of PPAR gamma 2/RXR alpha complexes. PPAR Res..

[64] Zeng ZP, Sun Z, Huang MF, Zhang WD, Liu J, Chen LQ (2015). Nitrostyrene derivatives act as RXR alpha ligands to inhibit TNF alpha activation of NF-kappa B. Cancer Res.

[65] Xu L, Zeng Z, Zhang W, Ren G, Ling X, Huang F (2017). RXRalpha ligand Z-10 induces PML-RARalpha cleavage and APL cell apoptosis through disrupting PML-RARalpha/RXRalpha complex in a cAMP-independent manner. Oncotarget.

[66] Deuster E, Jeschke U, Ye Y, Mahner S, Czogalla B (2017). Vitamin D and VDR in gynecological cancers-a systematic review. Int J Mol Sci..

[67] Craig TA, Benson LM, Tomlinson AJ, Veenstra TD, Naylor S, Kumar R (1999). Analysis of transcription complexes and effects of ligands by microelectrospray ionization mass spectrometry. Nat Biotechnol.

[68] Zhang H, Zhou R, Li L, Chen J, Chen L, Li C (2011). Danthron functions as a retinoic X receptor antagonist by stabilizing tetramers of the receptor. J Biol Chem.

[69] Chen L, Wang ZG, Aleshin AE, Chen F, Chen J, Jiang F (2014). Sulindac-derived RXRalpha modulators inhibit cancer cell growth by binding to a novel site. Chem Biol.

[70] Lu N, Liu J, Liu J, Zhang C, Jiang F, Wu H (2012). Antagonist effect of triptolide on AKT activation by truncated retinoid X receptor-alpha. PLoS One.

[71] Wang ZG, Chen L, Chen J, Zheng JF, Gao W, Zeng Z (2013). Synthesis and SAR study of modulators inhibiting tRXRalpha-dependent AKT activation. Eur J Med Chem.

[72] Wang GH, Jiang FQ, Duan YH, Zeng ZP, Chen F, Dai Y (2013). Targeting truncated retinoid X receptor-alpha by CF31 induces TNF-alpha-dependent apoptosis. Cancer Res.

[73] Chen F, Chen JB, Lin JC, Cheltsov AV, Xu L, Chen Y (2015). NSC-640358 acts as RXR alpha ligand to promote TNF alpha-mediated apoptosis of cancer cell. Protein Cell.

[74] Lemotte PK, Keidel S, Apfel CM (2010). Phytanic acid is a retinoid X receptor ligand. Eur J Biochem.

[75] Valerie V, Christina Z, Wurtz JM, Bourguet W, Kagechika H, Umemiya H (2014). A mutation mimicking ligand-induced conformational change yields a constitutive RXR that senses allosteric effects in heterodimers. EMBO J.

[76] Egea PF, Mitschler A, Rochel N, Ruff M, Chambon P, Moras D (2014). Crystal structure of the human RXRα ligand-binding domain bound to its natural ligand: 9-*cis* retinoic acid. EMBO J.

[77] Chen F, Liu J, Huang M, Hu M, Su Y, Zhang XK (2014). Identification of a new RXRalpha antagonist targeting the Coregulator-binding site. ACS Med Chem Lett.

[78] Burley SK, Berman HM, Christie C, Duarte JM, Feng Z, Westbrook J (2018). RCSB protein data Bank: sustaining a living digital data resource that enables breakthroughs in scientific research and biomedical education. Protein Sci A Publ Protein Soc.

[79] Burley SK, Berman HM, Kleywegt GJ, Markley JL, Nakamura H, Velankar S (2017). Protein data Bank (PDB): the single global macromolecular structure archive. Methods Mol Biol.

[80] Furnham N, Laskowski RA, Thornton JM (2013). Abstracting knowledge from the protein data Bank. Biopolymers.

[81] Rose PW, Bi C, Bluhm WF, Christie CH, Dimitropoulos D, Dutta S (2013). The RCSB protein data Bank: new resources for research and education. Nucleic Acids Res.

[82] Rose PW, Prlic A, Altunkaya A, Bi C, Bradley AR, Christie CH (2017). The RCSB protein data bank: integrative view of protein, gene and 3D structural information. Nucleic Acids Res.

[83] Berman HM, Kleywegt GJ, Nakamura H, Markley JL (2013). The future of the protein data Bank. Biopolymers.

[84] Osz J, McEwen AG, Poussin-Courmontagne P, Moutier E, Birck C, Davidson I (2015). Structural basis of natural promoter recognition by the retinoid X nuclear receptor. Sci Rep.

[85] Zhao Q, Chasse SA, Devarakonda S, Sierk ML, Ahvazi B, Rastinejad F (2000). Structural basis of RXR-DNA interactions. J Mol Biol.

[86] Sato Y, Ramalanjaona N, Huet T, Potier N, Osz J, Antony P (2010). The “phantom effect” of the Rexinoid LG100754: structural and functional insights. PLoS One.

[87] Kojetin DJ, Matta-Camacho E, Hughes TS, Srinivasan S, Nwachukwu JC, Cavett V (2015). Structural mechanism for signal transduction in RXR nuclear receptor heterodimers. Nat Commun.

[88] Bourguet W, Vivat V, Wurtz JM, Chambon P, Gronemeyer H, Moras D (2000). Crystal structure of a heterodimeric complex of RAR and RXR ligand-binding domains. Mol Cell.

[89] Washburn DG, Hoang TH, Campobasso N, Smallwood A, Parks DJ, Webb CL (2009). Synthesis and SAR of potent LXR agonists containing an indole pharmacophore. Bioorg Med Chem Lett.

[90] Wallace BD, Betts L, Talmage G, Pollet RM, Holman NS, Redinbo MR (2013). Structural and functional analysis of the human nuclear xenobiotic receptor PXR in complex with RXRalpha. J Mol Biol.

[91] Egea PF, Mitschler A, Rochel N, Ruff M, Chambon P, Moras D (2000). Crystal structure of the human RXRalpha ligand-binding domain bound to its natural ligand: 9-*cis* retinoic acid. EMBO J.

[92] Egea PF, Mitschler A, Moras D (2002). Molecular recognition of agonist ligands by RXRs. Mol Endocrinol.

[93] Scheepstra M, Andrei SA, de Vries RMJM, Meijer FA, Ma JN, Burstein ES (2017). Ligand dependent switch from RXR Homo- to RXR-NURR1 Heterodimerization. ACS Chem Neurosci.

[94] Zhang HT, Li L, Chen LL, Hu LH, Jiang HL, Shen X (2011). Structure basis of Bigelovin as a selective RXR agonist with a distinct binding mode. J Mol Biol.

[95] Bourguet W, Ruff M, Chambon P, Gronemeyer H, Moras D (1995). Crystal structure of the ligand-binding domain of the human nuclear receptor Rxr-alpha. Nature.

[96] Holmbeck SM, Foster MP, Casimiro DR, Sem DS, Dyson HJ, Wright PE (1998). High-resolution solution structure of the retinoid X receptor DNA-binding domain. J Mol Biol.

[97] Gampe RT, Montana VG, Lambert MH, Wisely GB, Milburn MV, Xu HE (2000). Structural basis for autorepression of retinoid X receptor by tetramer formation and the AF-2 helix. Genes Dev.

[98] Billas IM, Moulinier L, Rochel N, Moras D (2001). Crystal structure of the ligand-binding domain of the ultraspiracle protein USP, the ortholog of retinoid X receptors in insects. J Biol Chem.

[99] Nakano S, Yamada S, Okazaki S, Kakuta H, Tokiwa H. Crystal structure of RXR-LBD complexed with fluorescent ligand at 2.60 angstroms resolution. To be published.

