# Supplementary material for: Overview of the structure-based non-genomic effects of the nuclear receptor RXRα
Source: Cell Mol Biol Lett. 2018 Aug 7;23:36. doi: 10.1186/s11658-018-0103-3 (PMC6080560; doi:10.1186/s11658-018-0103-3)
Supplement: Supplementary file 1 — Table S1. The 3D structure of RXRα with compounds. (DOCX 1855 kb) [file 11658_2018_103_MOESM1_ESM.docx]

Additional file

Additional file 1: TableS1 The 3D structure of RXRα with compounds

| **PDB** | | | **Resolution** | | | **Released** | | | **Ref** | | **Description** | | | | **3D view** |  |  |  |
| --- | --- | --- | --- | --- | --- | --- | --- | --- | --- | --- | --- | --- | --- | --- | --- | --- | --- | --- |
| 1MVC | | | 1.9 | | | 2002-10-16 | | | [1] | Crystal structure of the human RXRα LBD bound to the synthetic agonist compound BMS 649 and a co-activator peptide | | | | 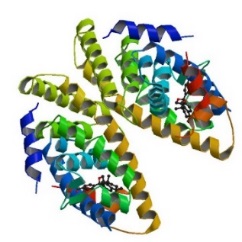 | |  |  |  |
| 2ZXZ | | | 3.0 | | | 2009-08-11 | | | [2] | Crystal structure of the human RXRα LBD bound to a synthetic agonist compound and a co-activator peptide | | | | 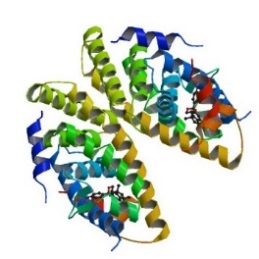 | |  |  |  |
| 2ZY0 | | | 2.9 | | | 2009-08-11 | | | [2] | Crystal structure of the human RXRα LBD bound to a synthetic agonist compound and a co-activator peptide | | | | 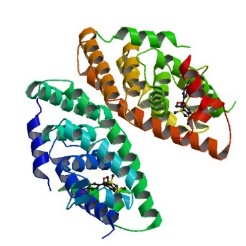 | |  |  |  |
| 3R5M | | | 2.8 | | | 2012-02-08 | | | [3] | Crystal structure of the RXRα LBD complexed with the agonist magnolol | | | | 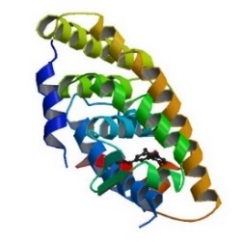 | |  |  |  |
| 4N8R | | | 2.03 | | | 2014-05-14 | | | [4] | Crystal structure of the RXRα LBD complexed with a synthetic modulator K-8008 | | | | 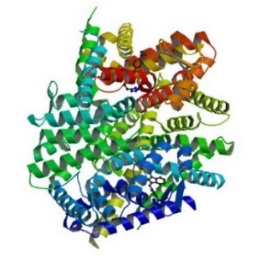 | |  |  |  |
| 3R29 | | | 2.9 | | | 2011-05-25 | | | [5] | Crystal structure of the RXRα LBD complexed with the co-repressor SMRT2 | | | | 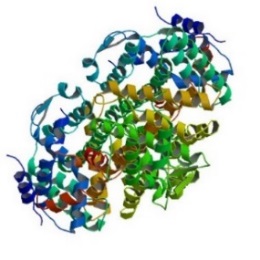 | |  |  |  |
| **3R2A** | | | 3.0 | | | 2011-05-25 | | | [5] | Crystal structure of the RXRα LBD complexed with the co-repressor SMRT2 and antagonist rhein | | | | 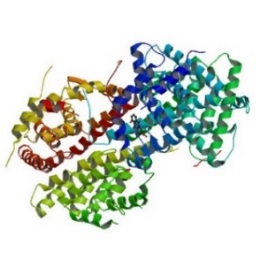 | |  |  |  |
| **3UVV** | | | 2.95 | | | 2012-04-18 | | | [6] | Crystal structure of the LBDs of the thyroid receptor RXR complexed with 3,3’,5 triiodo-L-thyronine and 9-*cis* retinoic acid | | | | 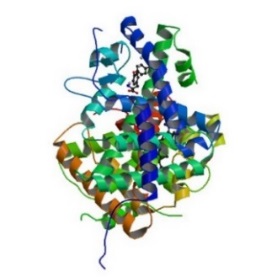 | |  |  |  |
| **4ZSH** | | | 1.8 | | | 2016-03-30 | | | [7] | The RXR LBD in complex with 9-*cis*-13,14-dihydroretinoic acid | | | | 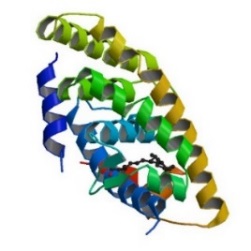 | |  |  |  |
| **3E94** | | | 1.9 | | | 2009-03-10 | | | [8] | Crystal structure of the RXRα LBD in complex with tributyltin and a co-activator fragment | | | | 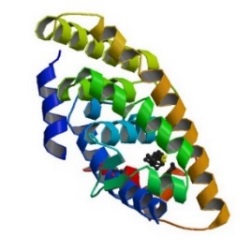 | |  |  |  |
| **3KWY** | | | 2.3 | | | 2010-01-19 | | | [9] | Crystal structure of the RXRα LBD in complex with triphenyltin and a co-activator fragment | | | | 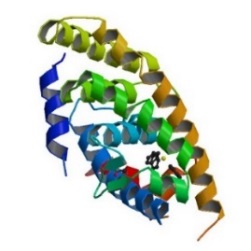 | |  |  |  |
| **3OAP** | | | 2.05 | | | 2010-11-17 | | | [10] | Crystal structure of the human RXRα LBD complex with 9-*cis* retinoic acid and the co-activator peptide GRIP-1 | | | | 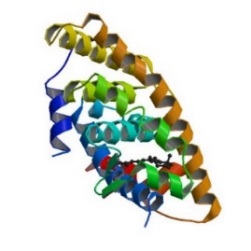 | |  |  |  |
| **2P1T** | | | 1.8 | | | 2007-10-09 | | | [11] | Crystal structure of the LBD of RXRα in complex with 3-(2’-methoxy)-tetrahydronaphtyl cinnamic acid and a fragment of the co-activator TIF-2 | | | | 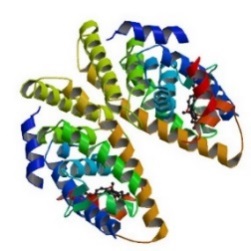 | |  |  |  |
| **2P1U** | | | 2.2 | | | 2007-10-09 | | | [11] | Crystal structure of the LBD of RXRα in complex with 3-(2’-ethoxy)-tetrahydronaphtyl cinnamic acid and a fragment of the co-activator TIF-2 | | | | 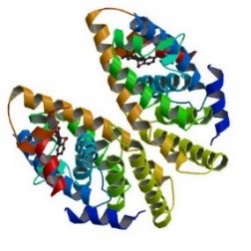 | |  |  |  |
| **2P1V** | | | 2.2 | | | 2007-10-09 | | | [11] | Crystal structure of the LBD of RXRα in complex with 3-(2’-propoxy)-tetrahydronaphtyl cinnamic acid and a fragment of the co-activator TIF-2 | | | | 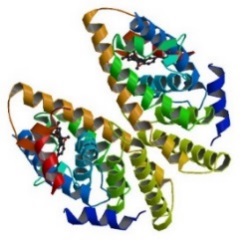 | |  |  |  |
| **4M8E** | | | 2.4 | | | 2014-01-22 | | | [12] | Crystal structure of human RXRα LBD complex with (S) 4-methyl 9cUAB30 co-activator peptide GRIP-1 | | | | 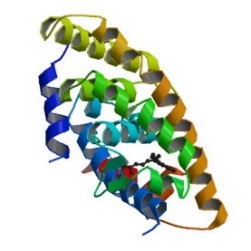 | |  |  |  |
| **4OC7** | | | 2.5 | | | 2014-10-08 | | | [13] | RARα in complex with (E)-3-(3’-allyl-6-hydroxy-[1,1’-biphenyl]-3-yl)acrylic acid and a fragment of the co-activator TIF2 | | | | 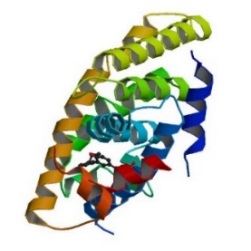 | |  |  |  |
| **4POH** | | | 2.3 | | | 2014-06-18 | | | [14] | Crystal structure of the human RXRα LBD complex with 8-methyl UAB30 and the co-activator peptide GRIP-1 | | | | 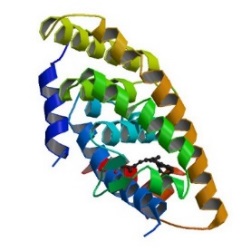 | |  |  |  |
| **4POJ** | | | 2.0 | | | 2014-06-18 | | | [14] | Crystal structure of the human RXRα LBD complex with 7-methyl UAB30 and the co-activator peptide GRIP-1 | | | | 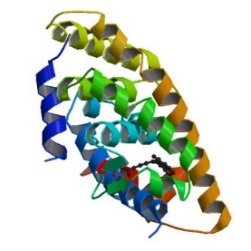 | |  |  |  |
| **4PP3** | | | 2.0 | | | 2014-06-18 | | | [14] | Crystal structure of the human RXRα LBD complex with 6-methyl UAB30 and the co-activator peptide GRIP-1 | | | | 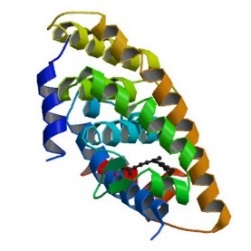 | |  |  |  |
| **4PP5** | | | 2.0 | | | 2014-06-18 | | | [14] | Crystal structure of the human RXRα LBD complex with 5-methyl UAB30 and the co-activator peptide GRIP-1 | | | | 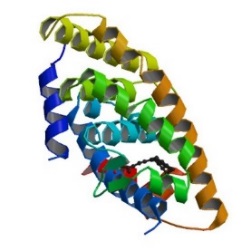 | |  |  |  |
| **4RFW** | | | 2.4 | | | 2015-09-16 | | | [15] | Crystal structure of the human RXRα LBD complex with 9cUAB70 and the co-activator peptide GRIP-1 | | | | 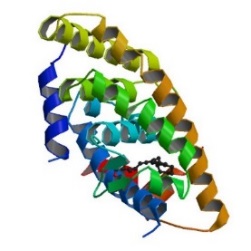 | |  |  |  |
| **4RMC** | | | 2.7 | | | 2015-09-16 | | | [15] | Crystal structure of the human RXRα LBD complex with 9cUAB76 and the co-activator peptide GRIP-1 | | | | 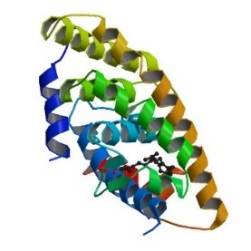 | |  |  |  |
| **4RMD** | | | 1.9 | | | 2015-09-23 | | | [15] | Crystal structure of the human RXRα LBD complex with 9cUAB110 and co-activator peptide GRIP-1 | | | | 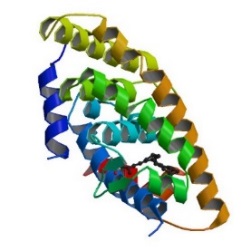 | |  |  |  |
| **4RME** | | | 2.3 | | | 2015-09-16 | | | [15] | Crystal structure of the human RXRα LBD complex with 9cUAB111 and co-activator peptide GRIP-1 | | | | 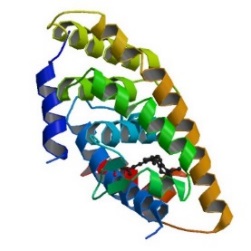 | |  |  |  |
| **3FUG** | | | 2.0 | | | 2009-05-12 | | | [16] | Crystal structure of the RXR LBD bound to the synthetic agonist 3-[4-Hydroxy-3-(3,5,5,8,8-pentamethyl-5,6,7,8-tetrahydronaphthalen-2-yl)-phenyl]acrylic acid | | | | 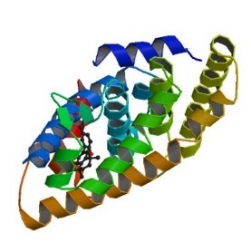 | |  |  |  |
| 5MMW | | | 2.7 | | | 2017-11-08 | | [17] | | | | Crystal structure of RXRα in complex with a synthetic honokiol derivative 6 and a fragment of the TIF2 co-activator | | 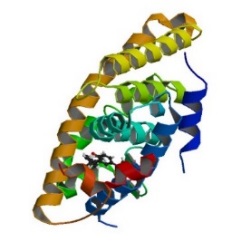 | | |  |  |
| 5MJ5 | | | 1.9 | | | 2017-11-08 | | | | [17] | | | Crystal structure of RXRα in complex with synthetic honokiol derivative 3 and a fragment of the TIF2 co-activator | 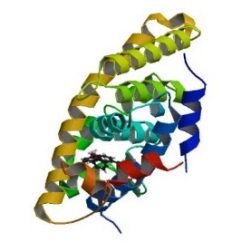 | | | | |
| 5MKJ | | | 2.5 | | | 2017-11-08 | | | | [17] | | | Crystal structure of RXRα in complex with synthetic honokiol derivative 9 and a fragment of the TIF2 co-activator | 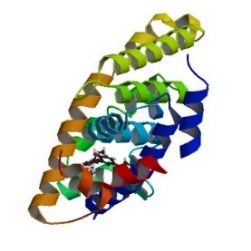 | | | | |
| 5MKU | | | 1.78 | | | 2017-11-08 | | | | [17] | | | Crystal structure of the RXRα in complex with synthetic honokiol derivative 4 and a fragment of the TIF2 co-activator | 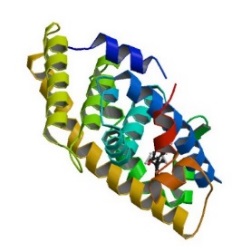 | | | | |
| 5EC9 | | | 2.3 | | | 2016-03-09 | | | | [18] | | | RXRα in complex with chiral dihydrobenzofuran benzoic acid 9a and a fragment of the co-activator TIF2 | 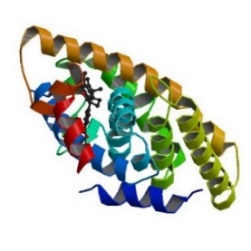 | | | | |
| 5LYQ | | | 2.17 | | | 2017-04-26 | | | | [19] | | | Crystal structure of the RXRα in complex with a synthetic spiroketal agonist and a fragment of the TIF2 co-activator | 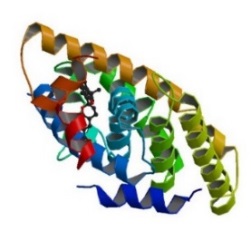 | | | | |

| **Supplement 2 The 3D structure of RXR**α **with DNA and/or nuclear receptor, and/or compounds** | | | | | | | | |
| --- | --- | --- | --- | --- | --- | --- | --- | --- |
| **PDB** | **Resolution** | | | **Released** | **Ref** | **Description** | | **3D view** |
| 3DZU | | 3.2 | 2008-10-28 | | [20] | Intact PPARγ-RXRα nuclear receptor complex on DNA bound with BVT.13, 9-*cis* retinoic acid and NCOA2 peptide | 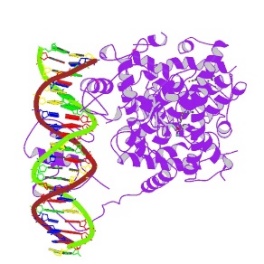 | |
| 3DZY | | 3.1 | 2008-10-28 | | [20] | Intact PPARγ-RXRα nuclear receptor complex on DNA bound with rosiglitazone, 9-*cis* retinoic acid and NCOA2 peptide | 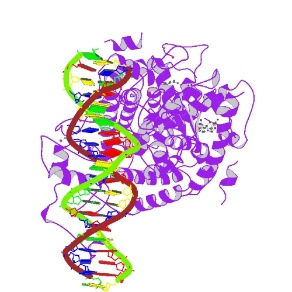 | |
| 3E00 | | 3.1 | 2008-10-28 | | [20] | Intact PPARγ-RXRα nuclear receptor complex on DNA bound with GW9662, 9-*cis* retinoic acid and NCOA2 peptide | 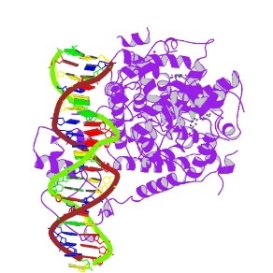 | |
| 4NQA | | 3.102 | 2014-02-26 | | [21] | Crystal structure of liganded hRXRα/hLXRβ heterodimer on DNA | 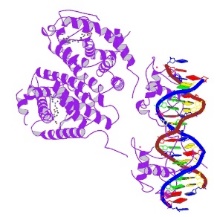 | |
| 1DSZ | | 1.7 | 2000-07-10 | | [22] | Structure of the RXR/RAR DNA-binding domain heterodimer in complex with the retinoic acid response element DR1 | 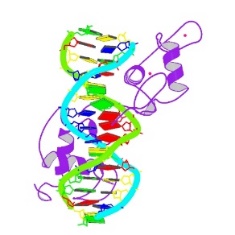 | |
| 1G5Y | | 2.0 | 2001-05-02 | | [23] | The 2.0 angstrom resolution crystal structure of the RXRα LBD tetramer in the presence of a non-activating retinoic acid isomer | 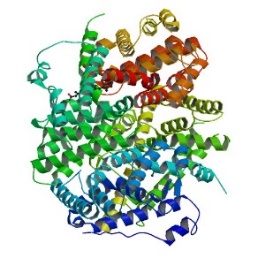 | |
| 1FM6 | | 2.1 | 2001-02-16 | | [24] | The 2.1 angstrom resolution crystal structure of the heterodimer of the human RXRα and PPARγ LBDs respectively bound with 9-*cis* retinoic acid and rosiglitazone and co-activator peptides | 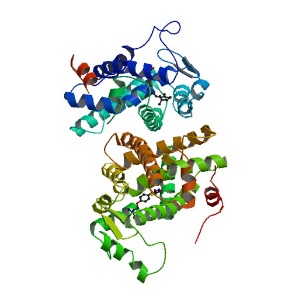 | |
| 1FM9 | | 2.1 | 2001-02-16 | | [24] | The 2.1 angstrom resolution crystal structure of the heterodimer of the human RXRα and PPARγ LBDs respectively bound with 9-*cis* retinoic acid and GI262570 and co-activator peptides | 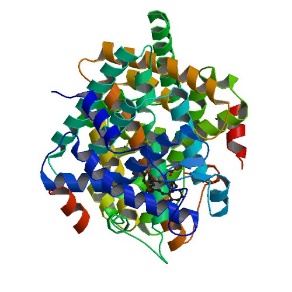 | |
| 3FAL | | 2.36 | 2009-04-14 | | [25] | Human RXRα and mouse LXRα complexed with retenoic acid and GSK2186 | 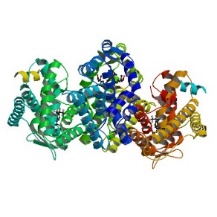 | |
| 3PCU | | 2.0 | 2011-11-16 | | [26] | Crystal structure of human RXRα LBD complexed with LX0278 and SRC1 peptide | 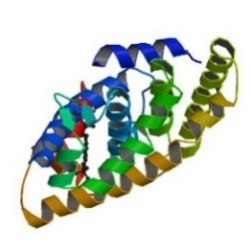 | |
| 4K4J | | 2.0 | 2013-11-13 | | [27] | Crystal structure of human RXRα LBD complex with 9cUAB30 and the co-activator peptide GRIP-1 | 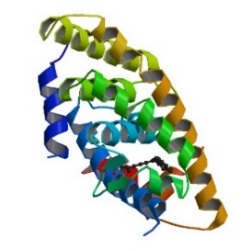 | |
| 4K6I | | 2.1 | 2013-11-13 | | [27] | Crystal structure of human RXRα LBD complex with Targretin and the co-activator peptide GRIP-1 | 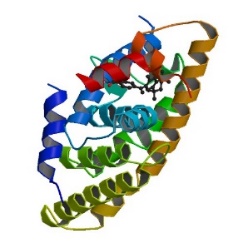 | |
| 4M8H | | 2.2 | 2014-01-22 | | [12] | Crystal structure of human RXRα LBD complex with (R)4-methyl 9cUAB30 and co-activator peptide GRIP-1 | 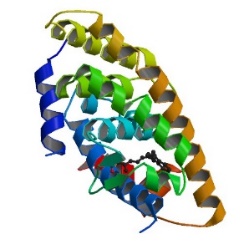 | |
| 1XDK | | 2.9 | 2004-11-09 | | [28] | Crystal structure of the RARβ/RXRα LBD heterodimer in complex with 9-*cis* retinoic acid and a fragment of the TRAP220 co-activator | 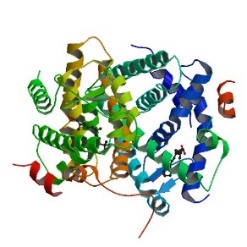 | |
| 1XLS | | 2.96 | 2004-12-28 | | [29] | Crystal structure of the mouse CAR/RXR LBD heterodimer bound to TCPOBOP and 9-*cis*-retanoic acid and a TIF2 peptide containing the third LXXLL motifs | 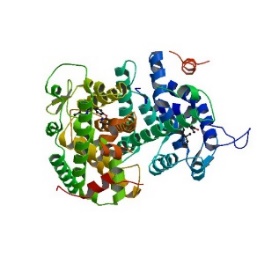 | |
| 1XV9 | | 2.7 | 2004-12-28 | | [30] | Crystal structure of CAR/RXR heterodimer bound with SRC1 peptide, fatty acid, and 5b-pregnane-3,20-dione | 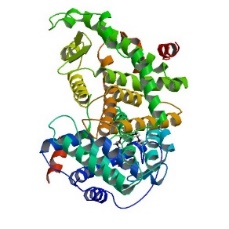 | |
| 1XVP | | 2.6 | 2004-12-28 | | [30] | Crystal structure of CAR/RXR heterodimer bound with SRC1 peptide, fatty acid and CITCO | 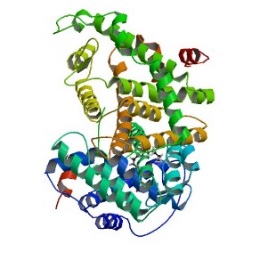 | |
| 1K74 | | 2.3 | 2001-12-05 | | [31] | The 2.3 Angstrom resolution crystal structure of the heterodimer of the human PPARγ and RXRα LBDs respectively bound with GW409544 and 9-*cis* retinoic acid and co-activator peptides | 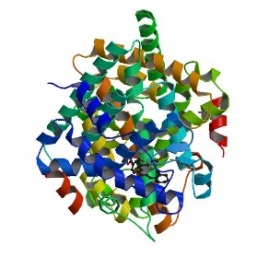 | |
| 1RDT | | 2.4 | 2004-11-09 | | [32] | Crystal structure of a new rexinoid bound to the RXRα LBD in the RXRα/PPARγ heterodimer | 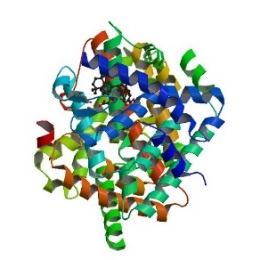 | |
| 5JI0 | | 1.98 | 2017-04-26 | | [33] | PPARγ–RXRα (S427F) heterodimer in complex with SRC-1, rosiglitazone and 9-*cis*-retanoic acid | 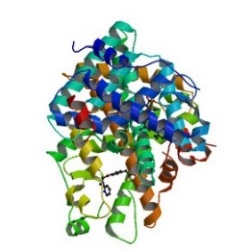 | |
| 5UAN | | 3.508 | 2017-10-18 | | [34] | Crystal structure of multi-domain RARβ–RXRα heterodimer on DNA | 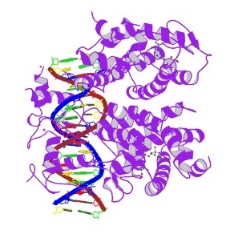 | |

**References**

1. Egea PF, Mitschler A, Moras D. Molecular recognition of agonist ligands by RXRs. Molecular endocrinology*.* 2002; 16:987–97.

2. Lippert WP, Burschka C, Gotz K, Kaupp M, Ivanova D, Gaudon C, et al. Silicon analogues of the RXR-selective retinoid agonist SR11237 (BMS649): chemistry and biology. ChemMedChem. 2009; 4:1143–52.

3. Zhang H, Xu X, Chen L, Chen J, Hu L, Jiang H, et al. Molecular determinants of magnolol targeting both RXRalpha and PPARgamma. PloS one. 2011; 6:e28253.

4. Chen L, Wang ZG, Aleshin AE, Chen F, Chen J, Jiang F, et al. Sulindac-derived RXRalpha modulators inhibit cancer cell growth by binding to a novel site. Chemistry & biology. 2014; 21:596-–07.

5. Zhang H, Chen L, Chen J, Jiang H, Shen X. Structural basis for retinoic X receptor repression on the tetramer. Journal of biological chemistry. 2011; 286:24593–98.

6. Putcha BD, Wright E, Brunzelle JS, Fernandez EJ. Structural basis for negative cooperativity within agonist-bound TR:RXR heterodimers. Proceedings of the National Academy of Sciences of the United States of America. 2012; 109:6084–87.

7. Ruhl R, Krzyzosiak A, Niewiadomska-Cimicka A, Rochel N, Szeles L, Vaz B, et al. 9-*cis*-13,14-Dihydroretinoic Acid Is an Endogenous Retinoid Acting as RXR Ligand in Mice. PLoS genetics. 2015; 11:e1005213.

8. le Maire A, Grimaldi M, Roecklin D, Dagnino S, Vivat-Hannah V, Balaguer P, et al. Activation of RXR-PPAR heterodimers by organotin environmental endocrine disruptors. EMBO reports. 2009; 10:367–73.

9. le Maire A, Bourguet W, Balaguer P. A structural view of nuclear hormone receptor: endocrine disruptor interactions. Cell Mol Life Sci. 2010; 67:1219–37.

10. Xia G, Boerma LJ, Cox BD, Qiu C, Kang S, Smith CD, et al. Structure, energetics, and dynamics of binding coactivator peptide to the human retinoid X receptor alpha ligand binding domain complex with 9-*cis*-retinoic acid. Biochemistry. 2011; 50:93–105.

11. Nahoum V, Perez E, Germain P, Rodriguez-Barrios F, Manzo F, Kammerer S, et al. Modulators of the structural dynamics of the retinoid X receptor to reveal receptor function. Proceedings of the National Academy of Sciences of the United States of America. 2007; 104:17323–28.

12. Desphande A, Xia G, Boerma LJ, Vines KK, Atigadda VR, Lobo-Ruppert S, et al. Methyl-substituted conformationally constrained rexinoid agonists for the retinoid X receptors demonstrate improved efficacy for cancer therapy and prevention. Bioorganic & medicinal chemistry. 2014; 22:178–85.

13. Scheepstra M, Nieto L, Hirsch AK, Fuchs S, Leysen S, Lam CV, et al. A natural-product switch for a dynamic protein interface. Angewandte Chemie. 2014; 53:6443–48.

14. Atigadda VR, Xia G, Desphande A, Boerma LJ, Lobo-Ruppert S, Grubbs CJ,et al. Methyl substitution of a rexinoid agonist improves potency and reveals site of lipid toxicity. Journal of medicinal chemistry. 2014; 57:5370–80.

15. Atigadda VR, Xia G, Deshpande A, Wu L, Kedishvili N, Smith CD, et al. Conformationally Defined Rexinoids and Their Efficacy in the Prevention of Mammary Cancers. Journal of medicinal chemistry. 2015; 58:7763–74.

16. Perez Santin E, Germain P, Quillard F, Khanwalkar H, Rodriguez-Barrios F, Gronemeyer H, et al. Modulating retinoid X receptor with a series of (E)-3-[4-hydroxy-3-(3-alkoxy-5,5,8,8-tetramethyl-5,6,7,8-tetrahydronaphthalen-2-y l)phenyl]acrylic acids and their 4-alkoxy isomers. Journal of medicinal chemistry. 2009; 52:3150–58.

17. Scheepstra M, Andrei SA, de Vries R, Meijer FA, Ma JN, Burstein ES, et al. Ligand Dependent Switch from RXR Homo- to RXR-NURR1 Heterodimerization. ACS chemical neuroscience. 2017; 8:2065–77.

18. Sunden H, Schafer A, Scheepstra M, Leysen S, Malo M, Ma JN, et al. Chiral Dihydrobenzofuran Acids Show Potent Retinoid X Receptor-Nuclear Receptor Related 1 Protein Dimer Activation. Journal of medicinal chemistry. 2016; 59:1232–38.

19. Scheepstra M, Andrei SA, Unver MY, Hirsch AKH, Leysen S, Ottmann C, et al. Designed Spiroketal Protein Modulation. Angewandte Chemie. 2017; 56:5480–84.

20. Chandra V, Huang P, Hamuro Y, Raghuram S, Wang Y, Burris TP, et al. Structure of the intact PPAR-gamma-RXR- nuclear receptor complex on DNA. Nature. 2008; 456:350–6.

21. Lou X, Toresson G, Benod C, Suh JH, Philips KJ, Webb P,et al. Structure of the retinoid X receptor alpha-liver X receptor beta (RXRalpha-LXRbeta) heterodimer on DNA. Nature structural & molecular biology. 2014; 21:277–81.

22. Rastinejad F, Wagner T, Zhao Q, Khorasanizadeh S. Structure of the RXR-RAR DNA-binding complex on the retinoic acid response element DR1. The EMBO journal. 2000; 19:1045–54.

23. Gampe RT, Jr., Montana VG, Lambert MH, Wisely GB, Milburn MV, et al. Structural basis for autorepression of retinoid X receptor by tetramer formation and the AF-2 helix. Genes & development. 2000; 14:2229–41.

24. Gampe RT, Jr., Montana VG, Lambert MH, Miller AB, Bledsoe RK, et al. Asymmetry in the PPARgamma/RXRalpha crystal structure reveals the molecular basis of heterodimerization among nuclear receptors. Molecular cell. 2000; 5:545–55.

25. Chao EY, Caravella JA, Watson MA, Campobasso N, Ghisletti S, Billin AN, et al. Structure-guided design of N-phenyl tertiary amines as transrepression-selective liver X receptor modulators with anti-inflammatory activity. Journal of medicinal chemistry. 2008; 51:5758–65.

26. Zhang Y, Zhang H, Yao XG, Shen H, Chen J, Li C, et al. (+)-Rutamarin as a dual inducer of both GLUT4 translocation and expression efficiently ameliorates glucose homeostasis in insulin-resistant mice. PloS one. 2012; 7(2):e31811.

27. Boerma LJ, Xia G, Qui C, Cox BD, Chalmers MJ, Smith CD, et al. Defining the communication between agonist and coactivator binding in the retinoid X receptor alpha ligand binding domain. The Journal of biological chemistry. 2014; 289:814–26.

28. Pogenberg V, Guichou JF, Vivat-Hannah V, Kammerer S, Perez E, Germain P, et al. Characterization of the interaction between retinoic acid receptor/retinoid X receptor (RAR/RXR) heterodimers and transcriptional coactivators through structural and fluorescence anisotropy studies. Journal of biological chemistry. 2005; 280:1625–33.

29. Suino K, Peng L, Reynolds R, Li Y, Cha JY, Repa JJ, et al.The nuclear xenobiotic receptor CAR: structural determinants of constitutive activation and heterodimerization. Molecular cell. 2004; 16:893–905.

30. Xu RX, Lambert MH, Wisely BB, Warren EN, Weinert EE, Waitt GM, et al. A structural basis for constitutive activity in the human CAR/RXRalpha heterodimer. Molecular cell. 2004; 16:919–28.

31. Xu HE, Lambert MH, Montana VG, Plunket KD, Moore LB, Collins JL, et al. Structural determinants of ligand binding selectivity between the peroxisome proliferator-activated receptors. Proceedings of the National Academy of Sciences of the United States of America. 2001; 98:13919–24.

32. Haffner CD, Lenhard JM, Miller AB, McDougald DL, Dwornik K, Ittoop OR, et al. Structure-based design of potent retinoid X receptor alpha agonists. Journal of medicinal chemistry. 2004; 47:2010–29.

33. Korpal M, Zhu P, Bloudoff K, Larsen NA, Fekkes P. PPARgamma-RXRalpha(S427F) heterodimer in complex with SRC-1, rosiglitazone, and 9-*cis*-retanoic acid. To be published.

34. Chandra V, Wu D, Li S, Potluri N, Kim Y, Rastinejad F. The quaternary architecture of RARbeta-RXRalpha heterodimer facilitates domain-domain signal transmission. Nature communications. 2017; 8:868.
